# A Review of the Antimicrobial Potential of *Musca domestica* as a Natural Approach with Promising Prospects to Countermeasure Antibiotic Resistance

**DOI:** 10.1155/2022/9346791

**Published:** 2022-12-30

**Authors:** Nurdjannah Jane Niode, Charles Kurnia Mahono, Felicia Maria Lolong, Merina Pingkan Matheos, Billy Johnson Kepel, Trina Ekawati Tallei

**Affiliations:** ^1^Department of Dermatology and Venereology, Faculty of Medicine, Sam Ratulangi University, Prof. Dr. R. D. Kandou Hospital Manado, Manado 9515, North Sulawesi, Indonesia; ^2^Department of Chemistry, Faculty of Medicine, Sam Ratulangi University, Manado 9515, North Sulawesi, Indonesia; ^3^Department of Biology, Faculty of Mathematics and Natural Sciences, Sam Ratulangi University, Manado 95115, North Sulawesi, Indonesia

## Abstract

Drug-resistant pathogens have become a serious public health concern worldwide considering the rapid emergence and distribution of new strains, which outpace the development of antimicrobial drugs. It is a complex and serious clinical problem that can cause an epidemic of a disease; consequently, numerous research studies are conducted to determine the solution to these problems, including the development of new antibiotics derived from natural sources such as insects. The housefly (*Musca domestica L.*), an insect known as a cosmopolitan pest, possesses several qualities that can ameliorate diseases; consequently, they can be used as a bioactive component in the development of medicines. These qualities include its potential as a source of antibacterial agents. The external surface components, wings, internal organs, and whole body extract of *M. domestica* can all contribute antimicrobial potential due to bioactive compounds they produce. This article discusses several antimicrobial properties of *M. domestica* that could be utilized for healthcare benefits.

## 1. Introduction

Drug-resistant pathogens present an ever-increasing global health threat due to the rapid emergence and distribution of new strains which is faster than the development of antimicrobial drugs [1–3]. This circumstance may result in the inappropriate or excessive utilization of antibiotics [4, 5]. There have been cases recorded of multidrug-resistant bacterial infection caused by *Escherichia coli* [6], methicillin-resistant *Staphylococcus aureus* (MRSA), *Streptococcus pneumoniae,* vancomycin-resistant *Enterococcus* (VRE)*, Pseudomonas aeruginosa, Acinetobacter baumannii,* and *Mycobacterium tuberculosis.* These antimicrobial-resistant superbugs have caused an alarming death rate of over 50% in certain regions [7]. It is a complex and serious clinical problem that can cause an epidemic of a disease, and hence several research studies are conducted to establish the solution to these problems, including the development of new antibiotics derived from nature, such as insects [6, 8, 9].

Insects and arthropods are considered a vast, unexplored, and underutilized source of potentially useful compounds for contemporary modern medicine [10]. They have a long history as a traditional therapy for humans and now have become more popular and are being developed for use in evidence-based practice [11, 12], in addition to becoming an important alternative therapy in the modern age in several countries such as India, Mexico, Korea, China, Spain, Brazil, Argentina, Ecuador, and several African countries [10, 13].

The housefly (*Musca domestica* L.) is among the Dipteran group and is a well-known cosmopolitan pest of livestock, poultry, and human dwellings. Houseflies are typically associated with humans or human activity [14]. Female houseflies lay countless eggs in animal waste, garbage, and other decaying matter [15]. The insect undergoes a complete life cycle, consisting of egg, larval, pupal, and adult stages, in 7 to 10 days [14]. They will live for 60 days the longest [15]. They prefer warm weather for optimal development, and hence they may thrive in the summer [16].

*M. domestica* is a vector for disease-causing bacteria due to its hopping and feeding behavior on a variety of pathogen-infested substrates [17, 18]. They also contribute to the spread of antibiotic-resistant bacteria, which can raise public health concerns [19]. However, contrary to the adult's existence as a vector for several diseases, the larvae of *M. domestica* has been used in the treatment of infectious diseases in Latin America and several other treatments for osteomyelitis, decubitus ulcers, eczema, malnutrition, and gastric cancer in China since the Qing and Ming dynasties until present days [6, 10, 13]. Due to the fact that scientific evidence has demonstrated that *M. domestica* larvae possess a variety of properties that can ameliorate diseases, they can be used as a source of bioactive component for pharmaceutical development [15, 20, 21]. These qualities include potential as antibacterial agents [10], even against bacteria that have developed multidrug resistance [6].

Housefly antimicrobial potential can come from the external surface components [15, 20, 22] and internal organs such as the digestive tract [6], hemolymph [23], and the insect's whole body extract [24, 25]. Therefore, this article focuses on the antimicrobial potentials that can be isolated from *M. domestica* and utilized for therapeutic purposes.

## 2. Microorganisms Contained in the Body Parts of *M. domestica*

Houseflies have a close association with microorganisms and their environments, especially at a crucial moment in each developmental stage [26]. The internal bacterial community of houseflies from various locations is similar and relatively stable, whereas the external bacterial community is affected by geography and habitat [27]. Several specific microbiota species isolated from various body parts of *M. domestica* are depicted in Table 1.

Bahrndorff et al. [28] reported that Firmicutes, Actinobacteria, Proteobacteria, and Bacteroidetes are phyla that dominate the entire microbiota of houseflies from 10 dairy farms in Denmark. In addition, Laziz et al. [22] isolated and identified 300 samples of houseflies (*M. domestica*) collected from different areas in Kirkuk City (Iraq) and found several species of Gram-positive and Gram-negative bacteria associated with body surface on the head, thorax, and abdomen (45.2%), right wing (35.7%), and left wing (19.1%). de Jonge et al. [29] revealed that *M. domestica*, both female and male, have a different population of bacteria in every segment of their digestive tracks.

The crop segment is abundant with *Streptococcus, Lactococcus, Leuconostoc*, and *Chishuiella*; the midgut segment is rich with *Delftia, Chryseobacterium, Acidovorax, Comamonas, Spirosoma,* and *Sphingomonas;* meanwhile other bacterial colonies found in both segments are *Pelagibacterium, Fructobacillus, Lactobacillus, Dyadobacter*, and *Novosphingobium*. The following bacterial phyla are present in accordance with the life cycle of the housefly: *Firmicutes* are abundant during the larval stages and are considered early colonizers, but as they mature into adults, *Proteobacteria* and *Bacteroidetes* take over. On the other hand, bacteria that exist throughout all stages are *Lactococcus*, *Lactobacillus*, and *Enterococcus*, while *Weissella* and *Chishuiella* were found in newly hatched larvae and adults, respectively.

Kanan et al. [30] successfully identified seven bacteria from houseflies sampled from Luwuk, Central Sulawesi, Indonesia, which previously had never been reported to be associated with flies. Nazari et al. [19] discovered that bacteria from the highest to lowest prevalence, respectively, are *Bacillus* spp. followed by *Staphylococcus* spp., *E. coli*, and *Enterococcus* spp. Yalli et al. [31] isolated *E. coli*, *Pseudomonas* spp., *Bacillus* spp., *Enterobacter* spp., *Staphylococcus* spp., *Salmonella* spp., *Proteus* spp., and *Klebsiella* spp. on the body surface of houseflies obtained from the kitchen, toilet, and room in Sokoto (Nigeria). Moreover, Nazni et al. [32] found *Bacillus* sp., *Coccobacillus* sp., *Staphylococcus* sp., *Micrococcus* sp., *Streptococcus* sp., *Acinetobacter* sp., *Enterobacter* sp., *Proteus* sp., *Klebsiella* sp., and yeast cell isolated from feces, vomitus, external surfaces, and internal organs of housefly collected from several regions in Malaysia.

## 3. Antimicrobial Potentials of *M. domestica*

**Figure 1:**
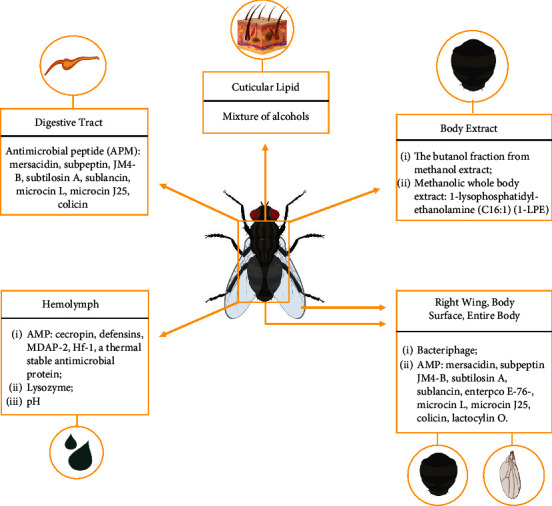
Antimicrobial components of *M. domestica* and their sources.

Secondary metabolites account for the majority of antimicrobials produced by microorganisms [9]. Insect physiology, such as resistance to pathogenic organisms, is influenced by numerous factors, including the gut microorganisms within the insect body [28]. *M. domestica* is known to have a diverse microbiome with antagonistic or antimicrobial properties that can impede the growth of pathogenic bacteria originating from the previous substrate [33]. Antagonistic activities from these bacteria may be associated with their abilities to secrete enzymes or compounds that function antagonistically and/or as an antimicrobial [18].  Table 2 and Figure 1 show various antimicrobial components belonging to *M. domestica,* which are derived from various parts of their body, and the bacteria that are the targets of these antimicrobials.

The production of early antimicrobial compounds by *M. domestica* larvae may protect the housefly from pathogenic microbes during the next developmental stages until it becomes an adult. These early antimicrobial compounds could be the primary antimicrobial compounds in their defense [43–45]. The presence of bacteria in the digestive system of a housefly indicates that *M. domestica* digestive tract produces antimicrobial compounds. These antimicrobial-producing bacteria in the wings and guts of insects are linked to their feeding behavior on microbe-contaminated substrates and stimulate the resistance response [18]. Laziz et al. [22] discovered that *B. subtilis* isolated from the right wing and body surface of *M. domestica* effectively inhibited the growth of *Pseudomonas* spp. *B. subtilis* plays an important role in the production of antibiotics, enzymes, and other secondary metabolites that possess a broad spectrum of antimicrobial activities against pathogenic microbes [46]. The right wing of *M. domestica* contains *B. subtilis* and *B. circulans* that can neutralize *E. coli* contaminated drinks due to their antibiotic effects. The enzymes and other secondary metabolites they produce can inhibit activities of several pathogenic microbes such as bacteria, fungi/yeasts, and parasites [20, 34]. Furthermore, the right wing contains bacteriophage, which is thought to produce endolysins (phage lysins), which causes bacterial cell lysis [20].

Another component of *M. domestica* that acts as a defense against microbes is hemolymph. This bactericidal effect from the hemolymph may counter-attack several bacteria including *Staphylococcus aureus, Staphylococcus epidermidis,* and *Pseudomonas aeruginosa* [42]. Hemolymph is a clear fluid (with or without greenish-yellow pigmentation) that contains very complex chemicals, mostly consisting of immune proteins and carbohydrates such as antimicrobial peptides (AMP), lysozyme, and agglutinins [23, 40, 47]. AMP is an innate immunity effector against bacteria, fungi, parasites, and viruses that possess several common properties such as cationicity, hydrophobicity, and amphipathicity for their antimicrobial activities [33]. A number of AMPs found in houseflies are cecropin [37], defensins [39, 48], MDAP-2 [40], and Hf-1 [10], as well as a cationic antimicrobial protein with a molecular weight of 16,315 D that is thermally stable and resistant to freezing and thawing [41]. AMPs are synthesized by immune and epithelial cells and secreted into hemolymph in response to infection and the presence of pathogenic bacteria [18]. The mechanisms of AMPs include binding to DNA, RNA, or intracellular protein [9] as well as inhibition of membrane protein and cell wall synthesis, altering the permeability of target cells [41]. Additionally, AMPs also induce apoptosis in eukaryotic cells and autolysis in bacterial cells and inhibit enzymes produced by some microbes, thereby reducing their virulence [9].

Another vital AMP is bacteriocin [35, 36]. *Bacillus* spp. found in the wings, digestives tract, and entire body of *M. domestica* produces bacteriocins such as mersacidin, subpeptin JM4-B, subtilosin A, and sublancin [35]. On the other hand, *Enterococcus* sp., which is found in the entire body and body surface, produces enterpco E-760 [35]. *Lactococcus* spp., which is found throughout the body, produces the lactic clinic Q. Body surfaces, the right wing, and the digestive tract harbor *E. coli* that produce microcin L, microcin J25, and colicin [35, 36].

The peritrophic matrix/membrane (PM protein) in a housefly's midgut plays a crucial role in preventing infection from outside microbes. The novel PM protein, MdPM-17, has been isolated from the housefly larvae. Several essential components of AMPs, including defensins, cecropins, and diptericin, are expressed by MdPM-17 recombinant protein silencing via RNA interference. This mechanism encourages the association between the MdPM-17 protein and the antibacterial response of houseflies [38]. Lysozymes are considered one of the innate immune effectors in flies that function in degrading pathogenic microbes [49]. As an antibacterial enzyme, lysozyme cleaves the *β*-1.4 glycosidic bond between N-acetylmuramic acid and N-acetylglucosamine, which are major components of the peptidoglycan structure of a bacterial cell wall [18]. Lysozyme activity is affected by several factors, including enzyme activity, pH level, and some effectors such as AMPs, which function to combat bacterial infections when the number is at an alarming level [44]. Lysozyme exerts its complex antibacterial defense strategies in response to infections [23]. Besides antibacterial proteins and carbohydrates in the hemolymph, it is possible that bactericidal potential is related to the acidity level (pH) through the increase of bacterial activities because of the decrease in pH level.

Additionally, other antimicrobial potentials of housefly can be seen from the butanol fraction obtained from ethanol extract of its larvae which demonstrate antibacterial activity against the methicillin-resistant *Staphylococcus aureus* (MRSA) and vancomycin-resistant *Enterococcus* (VRE strains) [25]. Housefly is also efficiently protected from infection by common pathogens inhabiting similar habitats through the association between the innate immunity mechanisms with mixtures of alcohols found in cuticular lipids of all stages (larvae, pupae, and adults) [14]. Moreover, 1-lysophosphatidylethanolamine (C_16:1_) (1-LPE) which is extracted from healthy uninfected last instar larvae can interfere with the growth of the Gram-positive bacteria (*Bacillus thuringiensis*) and the yeast *Saccharomyces cerevisiae* [24].

## 4. Conclusion

Evidence from a number of studies indicates that the common house fly, *Musca domestica*, possesses bioactive compounds with antimicrobial potential. These compounds originate from its organ components and the diverse microbiomes it harbors. The antagonistic activities of the diverse microbiome isolated from insect body parts are thought to be related to the ability to secrete enzymes or compounds that function as antimicrobial. Bacteriophage, AMP, lysozyme, pH, and alcohols contained in this insect have a direct or indirect bactericidal effect. However, a substantial amount of research is still required to investigate and develop the antimicrobial potentials of houseflies.

## Figures and Tables

**Table 1:** The type of bacteria isolated from housefly *M. domestica*.

Body part	Isolated microorganisms	References
Entire body	(i) Actinobacteria (*Microbacterium* spp.)	Bahrndorff et al. [28]
	(ii) Bacteroidetes/Bacteroidota (*Apibacter* spp., *Chishuiella* spp., *Chryseobacterium* spp., *Moheibacter* spp., *Spirosoma* spp., *Sphingobacterium* spp.)	de Jonge et al. [29]
	(iii) Firmicutes (*Bacillus* spp., *Enterococcus* spp., *Lactobacillus* spp., *Lactococcus* spp., *Leuconostoc* spp., *Weissella* spp.)	
	(iv) Fusobacteria	
	(v) Lentisphaerota/Lentisphaerae	
	(vi) Proteobacteria (*Acidovorax* spp., *Alcaligenes* spp., *Brevundimonas* spp., *Delftia* spp., *Klebsiella* spp., *Ochrobactrum* spp., *Paenochrobactrum* spp., *Pseudochrobactrum* spp., *Pseudomonas s*pp., *Stenotrophomonas* spp.)	
	(vii) Saccharibacteria (formerly known as TM7)	
	(viii) Spirochaetota/Spirochaetes	
	(ix) Tenericutes	

Right wing	(i) Actinobacteria (*Micrococcus luteus*)	Laziz et al. [22]
	(ii) Bacteroidetes/Bacteroidota (*Sphingobacterium* sp.)	Kanan et al. [30]
	(iii) Firmicutes (*Bacillus subtilis*, *Staphylococcus aureus*, *S. xylosus*)	
	(iv) Proteobacteria (*Acinetobacter* spp., *Brucella melitensis, Escherichia coli*, *Klebsiella oxytoca, Proteus vulgaris*, *P. fluorescens*)	

Left wing	(i) Actinobacteria (*Bifidobacterium minimum*)	Laziz et al. [22]
	(ii) Bacteroidetes/Bacteroidota (*Sphingobacterium* sp.)	Kanan et al. 2020 [30]
	(iii) Firmicutes (*Bacillus* spp., *Aerococcus viridans*, *Streptococcus iniae*	
	(iv) Proteobacteria (*Acinetobacter* spp., *Alcaligenes faecalis*, *Brucella melitensis* bv.1, *Enterobacter asburiae*, *P. fluorescens*)	

Body surfaces	(i) Firmicutes (*Bacillus cereus*, Coagulase-negative *Staphylococcus*, *Enterococcus* spp., S*. aureus*, S*. saprophyticus*)	Laziz et al. [22]
		Nazari et al. [19]
		Yalli et al. [31]
	(ii) Proteobacteria (*E. coli*, *Citrobacter* spp., *Enterobacter* spp., *Haemophilus ducreyi*, *Hafnia alvei*, *Klebsiella* spp., *K. oxytoca*, *Proteus* spp., *P. aeruginosa, Pseudomonas* spp., *Serratia fonticola*)	Nazni et al. [32]

Digestive tract	(i) Firmicutes (*Bacillus* sp.)	Nazni et al. [32]
	(ii) Proteobacteria (*Enterobacter* spp., *E. coli*, *Klebsiella* spp., *Proteus* spp.)	

**Table 2:** Antimicrobial components of *M. domestica* microbiome.

Body parts	Microbiome metabolites	Inhibited bacteria	References
Right wing, body surface, entire body	(i) Bacteriophage	(i) *Pseudomonas* spp.	(i) Laziz et al. [22]
	(ii) Antimicrobial peptide (AMP): mersacidin, subpeptin JM4-B, subtilosin A, sublancin, enterpco E-760, microcin L, microcin J25, colicin, lactocyclicin Q	(ii) *E. coli*	(ii) Claresta et al. [20]; Zhao et al. [34]
		(iii) *MRSA, Streptococcus pyogenes, S. agalactiae, *and *S. pneumoniae*	(iii) Simons et al. [35]
		(iv) *S. aureus, S. faecalis, Salmonella* sp., and *Shigella flexneri*	(iv) Yang et al. [36]
		(v) *Enterococcus faecalis *and *Listeria monocytogenes*	
		(vi) *Yersinia* spp., *Campylobacter* spp. *Staphylococcus* spp. *Listeria monocytogenes*	
		(vii) *Pseudomonas aeruginosa*	
		(viii) *Lactoccocus* spp.	

Hemolymph	(i) AMP: cecropin, defensins, Hf-1, MDAP-2, a thermal stable antimicrobial protein	*S. aureus, S. epidermidis,* and *P. aeruginosa*	(i) Liang et al. [37]; Wang et al. [38]; Dang et al. [39]; Hou et al. [10]; Pei et al. [40]; Hao et al. [41]
	(ii) Lysozyme		(ii) Kawasaki and Andoh [42]

Body extract	(i) The butanol fraction obtained from ethanol	(i) MRSA and VRE strains	(i) Park et al. [25]
	(ii) Methanolic whole body extract: 1-lysophosphatidylethanolamine (C_16:1_) (1-LPE)	(ii) *B. thuringiensis* and *Saccharomyces cerevisiae*	(ii) Meylaers [24]

Cuticular lipid	Mixtures of alcohols	*Rhodococcus equi, Candida lipolytica, C. tropicalis*	(i) Gołêbiowski [14]

Digestive track	AMP: mersacidin, subpeptin JM4-B, subtilosin A, sublancin, microcin L, microcin J25, and colicin	(i) *MRSA, Streptococcus pyogenes, S. agalactiae, *and* S. pneumoniae*	(i) Simons et al. [35]
		(ii) *S. aureus, S. faecalis, Salmonella* sp., and *Shigella flexneri*	(ii) Yang et al. [36]
		(iii) *Enterococcus faecalis* and *Listeria monocytogenes*	
		(iv) *E. coli, Salmonella enterica, Shigella* spp., and *Pseudomonas aeruginosa*	

## Data Availability

The research data are presented in the article. These data are publicly available and accessible online. Detailed sources are provided in References of the article.

## Abbreviations

AMPs: Antimicrobial peptides
LPE: Lysophosphatidylethanolamine
MRSA: Methicillin-resistant *Staphylococcus aureus*
PM: Peritrophic matrix/membrane
VRE: Vancomycin-resistant *Enterococcus.*
